# Osteoarthritis in a fifty-year-old patient with unfused skeleton due to undiagnosed coexisting cleidocranial and spondyloepiphyseal dysplasia – Case report

**DOI:** 10.1016/j.amsu.2022.104066

**Published:** 2022-06-28

**Authors:** Dr Mahum Zaidi, Sareema Eman Akhtar, Saad Shakil, Dr Muhammad Wasif, Dr Rabiya Siraj, Talal Almas, Joshua Ramjohn, Maha Alkhattab, Reema Ahmed, Tushar Thakur

**Affiliations:** a Radiology Department, Liaquat National Hospital, Karachi, Pakistan; b Ziauddin Medical College, Karachi, Pakistan; c ENT / Head and Neck Surgery, Dr. Ziauddin University Hospital, Karachi, Pakistan; d RCSI University of Medicine and Health Sciences, 123 St. Stephen's Green, Dublin 2, Ireland; e Sligo University Hospital, Sligo, Ireland; f Galway University Hospital, Galway, Ireland; g National University of Ireland, Galway, Ireland

**Keywords:** Cleidocranial dysplasia (CCD), Spondyloepiphyseal dysplasia (SED), Skeletal dysplasia, Radiology, Xray

## Abstract

**Background:**

Skeletal dysplasia's cause significant neurological symptoms and disrupt the development of many bones and cartilages in the body. Skeletal dysplasia, although a common presentation in paediatric population, rarely presents in older age group.

**Case presentation:**

This case presents a unique incidental finding of skeletal dysplasia in a fifty-year-old male patient who presented with osteoarthritis. Eventual workup uncloaked the presence of cleidocranial dysplasia and spondyloepiphyseal dysplasia. The patient in this case had both dysplasias at the same time.

**Discussion:**

Cleidocranial dysplasia and Spondyloepiphyseal dysplasia are two uncommon autosomal dominant dysplasia's that are often diagnosed in early life and can have serious consequences, including death. It is critical to diagnose a child early in life. Radiology findings from a thorough skeletal examination aid in the early detection of numerous dysplasia's, which helps improving quality of life and allowing for effective treatment.

**Conclusion:**

The novelty of our presented case lies in the rare presentation of CCD and SED occurring concurrently at an older age with accompanying collateral abnormalities usually emerging more commonly in infants. Early diagnosis is thus essential for optimal management.

## Highlights

• Skeletal dysplasia is a heterogeneous group of disorders which are genetic in nature and comprise around 400 disorders.
• In the case presented, radiological findings revealed two very rare skeletal dysplasia in a fifty year old patient which were cleidocranial dysplasia as well spondyloepiphyseal dysplasia.
• This is of particular importance as these are commonly present in neonates and infants but in this case, it was an incidental finding in a patient undergoing radiological investigations for osteoarthritis.

## 1 Introduction

Skeletal dysplasia is a heterogeneous group of disorders which are genetic in nature and comprise around 400 disorders. These disorders also known as achondroplasia, primarily affect the growth of bones and cartilages leading to its abnormal shape and size [1]. These disorders have been further categorized into those that involve either the epiphysis, metaphysis, the spine or a mix of these three [2]. The overall worldwide incidence of skeletal dysplasia is reported to be 1 case in 4000–5000 live births. These disorders manifest rarely in the childhood period and are usually evident in infancy or newly born children. Around 13% of the infants are found to be stillborn at birth with evident skeletal dysplasia and 4% suffer death during the perinatal period. As reported earlier, 9.1 per 1000 infants are affected by skeletal dysplasia who die perinatally [1]**.**

An autosomal dominant genetic trait known as Cleidocranial Dysplasia (CCD) is a rare skeletal disorder that is usually present at birth. The prevalence of this disorder at birth equals to 1 in million which highlights the rarity of the disorder. Affecting both the male and female gender equally, medical literature reports only one thousand cases till date. The link of this genetic disorder has been found to be a mutation in Core-Binding Factor Alpha 1 (CBFA1) gene [3]. Another rare inherited congenital disorder known as Spondyloepiphyseal Dysplasia (SED) is an autosomal dominant disorder that usually commences prenatally. Congenital dwarfism with a short trunk and epiphyseal dysplasia in the long bone and vertebral bodies characterizes this disorder [4]. The incidence of this disease is not exactly known but is estimated to be around 1 in 100,000 live births. This disorder is linked to the mutation in type 2 collagen (COL2A1) gene [5]. Although the concomitant presence of both dysplasias is a rare occurrence, its incidence remains very low, suggesting that the economical duress that it exerts on healthcare setups remains nominal.

In the case presented, radiological findings revealed two very rare skeletal dysplasia in a fifty year old patient which were cleidocranial dysplasia as well spondyloepiphyseal dysplasia. This is of particular importance as these are commonly present in neonates and infants but in this case, it was an incidental finding in a patient undergoing radiological investigations for osteoarthritis.

## 2 Case report

**Fig. 1:**
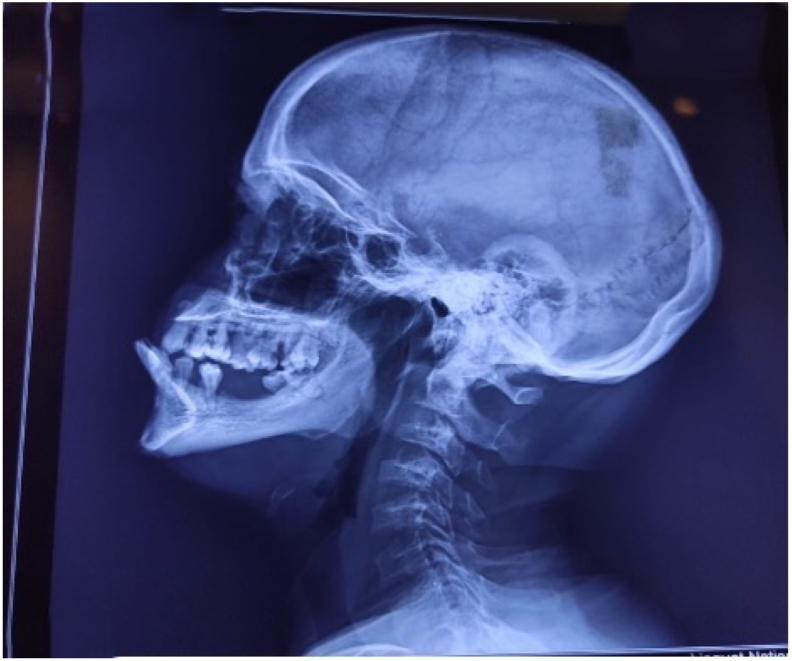
Xray skull lateral view showing absent frontal sinuses, hypoplastic nasal bridge, relative mandible prognathism which are features of cleidocranial dysplasia. Coexisting cervical platyspondyly is a feature of SED. (original image).

**Fig. 2:**
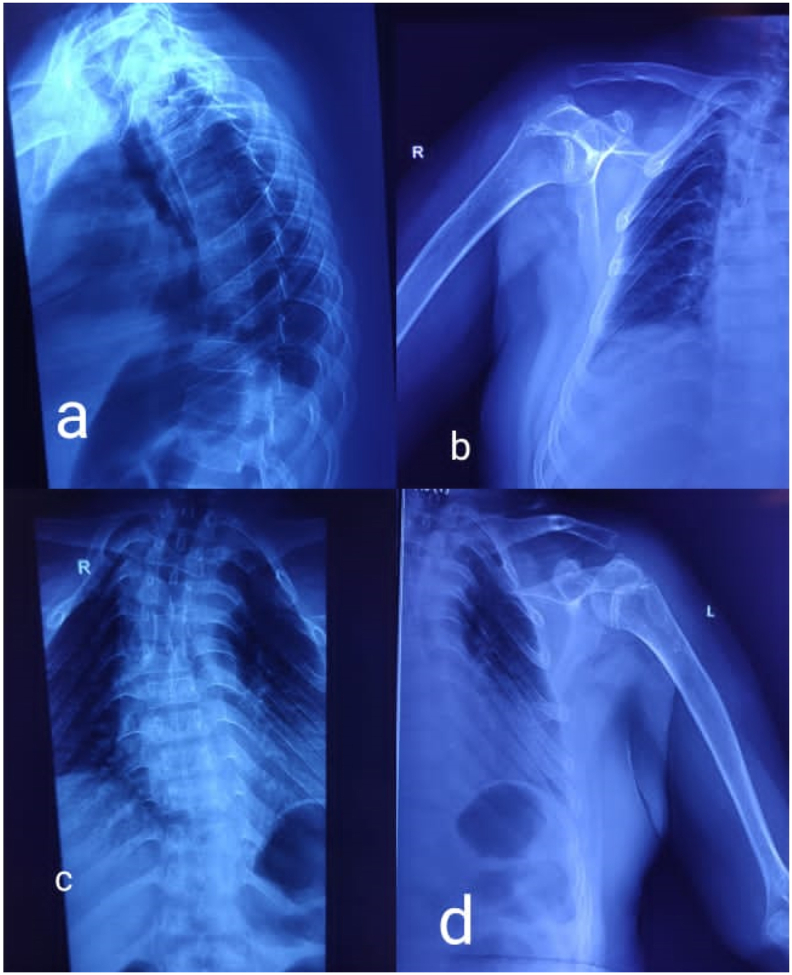
Xray skeletal survey showing a) dorsal platyspondyly b) and d) showing resorption of lateral end of clavicle with narrow funnel shaped thorax and unfused humeral epiphysis, c) scoliosis of dorsal spine. (original image).

**Fig. 3:**
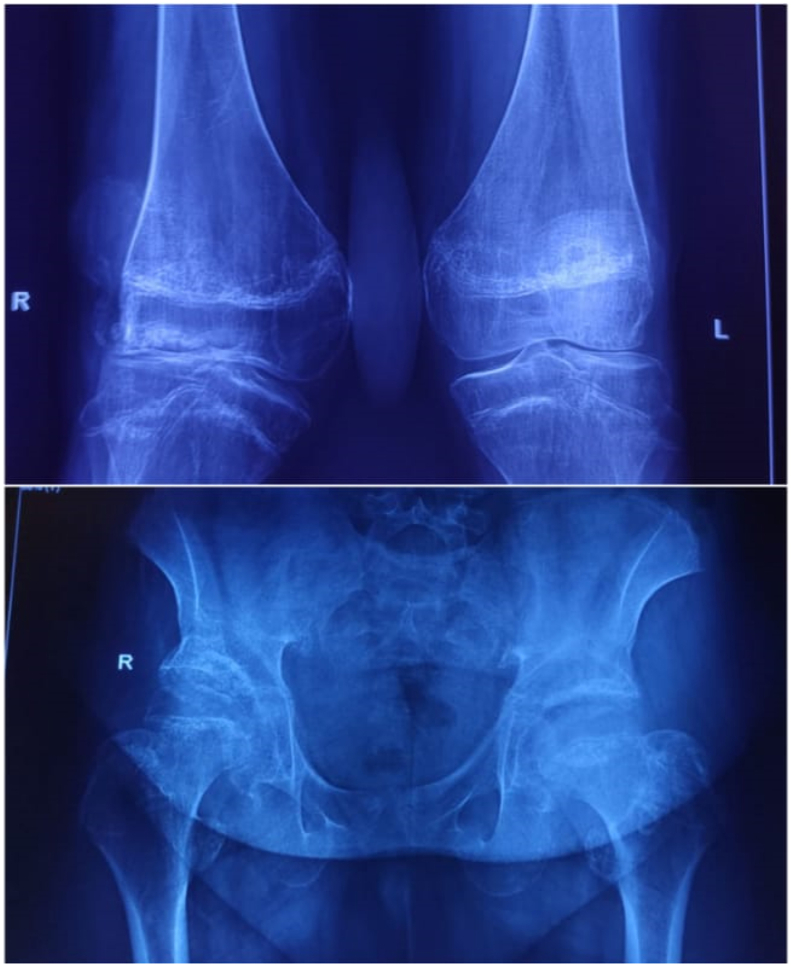
Xray knee and pelvis showing unfused skeleton with reduced tibiofemoral and hip joint space bilaterally and genu valgum deformity. (original image).

A 50 year old male presented to the rheumatology OPD with complaints of progressive bilateral knee pain for last 1 year for which he was advised X-Ray which showed reduced joint space in the medial compartment of bilateral tibiofemoral joint and osteophytes along its articular margins representing osteoarthritic changes. However, this patient was also incidentally found to have unfused skeleton on X-ray knee which is unusual at the age of fifty years. Therefore, a skeletal survey was advised to look for any congenital skeletal dysplasia regarding which the patient was unaware. The patient had a relatively short height of around 5 feet, his intelligence quotient was normal and he was leading a normal life until he developed pain one year back which was progressive in nature. The skeletal survey done after one week revealed a variety of unusual findings which included absent frontal sinuses, hypoplastic nasal bridge, relative prognathism however the skull size and sutures were normal. There was minimal resorption of the lateral ends of clavicles and a narrow bell-shaped thorax however the scapula and ribs were normal. All these features were corresponding with the diagnosis of Cleidocranial Dysplasia however there were certain features appreciated in skeletal survey like presence of platyspondyly in the whole spine and persistence of unfused skeleton in all long bones to the age of fifty years which was in turn suggestive of a co-existing Spondyloepiphyseal Dysplasia. Fig. 1, Fig. 2, Fig. 3 show the radiological findings of this patient. Osteoarthritis is a known complication of Spondyloepiphyseal dysplasia [11]. Written informed consent was obtained from the patient for publication of this case report and accompanying images. A copy of the written consent is available for review by the Editor-in-Chief of this journal on request. The present case was reported in line with the SCARE 2020 criteria [11].

## 3 Radiological images

The patient continues to do well to date, with regular six-monthly skeletal survey follow-ups scheduled to monitor disease progression.

## 4 Discussion

Skeletal dysplasia arises from the genetic mutation inherited from parents to their children leading to its vast effect on the growth and development of bones and cartilage. Achondroplasia is the most frequently occurring skeletal dysplasia with mutation in Fibroblast growth factor receptor 3 (FGFR3) gene. Other prevalent skeletal dysplasia includes osteogenesis imperfecta, hypochondroplasia, thanatophoric dysplasia, campomelic dysplasia and achondrogenesis. However, amongst them the prevalence of CCD and SED remains rare [6]. The key diagnostic components of the disease involve clinical examination with a detailed family history and radiological imaging. The principal measurements while evaluating the patient clinically involves the anthropometric measures. Radiological imaging includes anterior, posterior and lateral views of the bones.

SED predominantly comprises vertebrae and epiphyseal centres resulting in characteristic dwarfism with inordinate short trunk [7]. In addition to dwarfism other clinically characteristic features include scoliosis (sideway curvature of spine), cox vara, femoral head hypoplasia and flat vertebral bodies. Many other people with different disorders including Morqio's disease, Dyggyve-Melchior-Clausen dysplasia and Kniest-Stickler dysplasia are considered potential for differential diagnosis [4]. Other symptoms of this disorder include back ache, height of 35.5–49 inches, common neck problems due to unprogressive vertebrae, common involvement of retina as retinal detachment and hip pain due to arthritis [8]. Current literature detected os odontoideum in almost all patients out of whom 35–60% were found to have atlanto-axial instability. Among individuals evaluated, the onset of spinal cord symptoms ranged from childhood to patients of 40 years of age [4].

CCD classically affects children under five years of age. There are multiple complications associated, most common complications being Genua valga and pes planus amongst many others including respiratory, dental and auditory problems such as otitis media and sinus infections [9]**.** Short height, narrow sloped shoulders, unusual facial features are characteristics of cleidocranial dysplasia. Premature closure of the soft spot on the head (coronal), delayed closure of the gap between the bones of the skull (fontanels), thin and irregularly shaped pelvic and pubic bones, and deformations in the chest are some of the major symptoms (thoracic region) [3]. The diagnosis is classically made on the basis of physical examination and X-Rays [10]**.**

## 5 Limitations

The present paper reports a single case from one centre. As such, it represents a very limited scope of evidence and elucidates a single-centre experience with managing a rare radiological pathology. The diagnostic and therapeutic approached adopted in the present case might therefore not be generalisable to the wider audience. Additionally, there is an unmet need for larger, multi-centric studies with larger data sets to ascertain the true incidence, management, and prognosis of the aforesaid ailment.

## 6 Conclusion

The novelty of our presented case lies in the rare presentation of CCD and SED occurring concurrently at an older age with accompanying collateral abnormalities usually emerging more commonly in infants. Early diagnosis requires efficient and timely general physical examination and early recognition of clinical features.

## Ethical approval

Ethical approval letter from the head of department has been attached to the cover letter.

## Sources of funding

NA.

## Author contribution

Mahum Ziadi: Study design and writing of case report body. Also reviewed the article. Sareema Eman Akhtar: Literature search. Writing of manuscript introduction and discussion. Saad Shakil: Literature search. Writing of manuscript introduction and discussion. Muhammad Wasif: Supervised and reviewed the article. Rabiya Siraj: Supervised and reviewed the article. Talal Almas: Supervised and reviewed the article. Joshua Ramjohn: Supervised and reviewed the article. Maha Alkhattab: Supervised and reviewed the article. Tushar Thakur, Reema Ahmed: Revised the manuscript in line with reviewer comments. The manuscript has been read and approved by all authors, requirements of authorships have been met and each author believes that the manuscript represents honest work.

## Consent

Written informed consent was obtained from the patient for publication of this case report and accompanying images. A copy of the written consent is available for review by the Editor-in-Chief of this journal on request.

## Registration of research studies

1. Name of the registry: NA
2. Unique Identifying number or registration ID: NA
3. Hyperlink to your specific registration (must be publicly accessible and will be checked): NA

## Guarantor

Talal Almas RCSI University of Medicine and Health Sciences 123 St. Stephen's Green Dublin 2, Ireland Talalalmas.almas@gmail.com.

## Provenance and peer-review

Not commissioned, externally peer-reviewed.

## Disclosures

N/A.

## Patient consent

Written informed consent was obtained from the patient for publication of this case report and accompanying images. A copy of the written consent is available for review by the Editor-in-Chief of this journal on request.

## Declaration of competing interest

NA.
